# Vaginal lavage fluid can serve as a reliable method for early screening of endometrial cancer: a proof-of-concept study

**DOI:** 10.3389/fonc.2025.1736317

**Published:** 2026-01-12

**Authors:** Yibing Li, Ping Ren, Jiazhen Huang, Shuang Tan, Ning Wang

**Affiliations:** Department of Obstetrics and Gynecology, The Second Affiliated Hospital of Dalian Medical University, Dalian, Liaoning, China

**Keywords:** ctDNA, early screening, endometrial cancer, high-throughput sequencing, vaginal lavage fluid

## Abstract

**Background:**

Endometrial cancer (EC) is one of the malignant tumors in the female reproductive system, and effective screening is urgently needed to reduce mortality. At present, the main screening method for endometrial cancer is ultrasound combined with curettage and pathological examination. We plan to explore a non-invasive, convenient, and low-cost new screening method for EC, providing a more convenient way for early screening of EC.

**Methods:**

We included a total of 11 patients with stage IA EC, and collected 8-10ml of blood, pathological paraffin sections, and vaginal lavage fluid for each patient. The collection of vaginal lavage fluid was performed before the patient’s curettage or hysteroscopy for pathological examination. And perform high-throughput sequencing and further analysis on it.

**Results:**

In 11 cases with matched tumor and blood tissues, the extracted ctDNA concentration was significantly higher in vaginal lavage fluid than in plasma. The average and median MAF of different samples from paired tissue patients were significantly higher in vaginal lavage supernatant and sediment than in plasma. The types of genomic changes in the supernatant of vaginal lavage fluid are relatively consistent with those in the sediment and tumor tissue samples, but significantly different from those in the plasma. In addition, among the six most frequently mutated genes in this study, the consistency between vaginal lavage fluid supernatant and vaginal lavage fluid precipitation and tumor tissue genomic changes was significantly higher than in plasma. According to the results of 11 cases that provided tumor tissue samples, the genomic changes detected between the supernatant or precipitate of vaginal lavage fluid and tumor tissue were significantly consistent compared to those detected between tumor tissue and plasma. The potential targets for targeted treatment of endometrial cancer (KRAS, PIK3CA, MLH1, MSH6, POLE, PTEN) showed more significant changes in the supernatant of vaginal lavage fluid.

**Conclusion:**

The supernatant/precipitate of vaginal lavage fluid has significant clinical significance for early screening of endometrial cancer and may also have potential clinical value in guiding targeted therapy and evaluating prognosis. Vaginal lavage fluid gene sequencing may serve as a new method for initial screening of endometrial cancer.

## 1 Introduction

Endometrial cancer (EC) is one of the malignant tumors in the female reproductive system. In the United States, there will be 69120 new cases in 2025, ranking fourth after breast cancer, lung & bronchus cancer and colon & rectum cancer, and 13860 new deaths in 2025, ranking fifth (1) Therefore, effective screening for EC is urgently needed to reduce mortality. At present, the main screening method for endometrial cancer is ultrasound combined with curettage and pathological examination. Curettage is an invasive procedure with significant pain and high screening costs. Therefore, we plan to explore a non-invasive, convenient, and low-cost new screening method for EC, providing a more convenient way for early screening of EC.

Liquid biopsy technology plays an important role in the early diagnosis and screening of various tumors, and has been applied in various types of tumors (2–11).Such as lung cancer (2–4), bile duct cancer (5, 6), pancreatic cancer (7), liver cancer (8), breast cancer (9), bladder cancer (10) and colorectal cancer (11) The vast majority of cell-free DNA (cfDNA) is released from hematopoietic cells, while a small portion is released from cancer cells (12) Therefore, the characteristics of cfDNA are closely related to early tumor development. In the study of liquid biopsy techniques related to EC, it is feasible to detect circulating tumor cells in ovarian vein blood samples of patients undergoing laparoscopic surgery for early EC (13) In surgical stratification and follow-up of patients with moderate, high-risk, and advanced EC, liquid biopsy techniques can be used to evaluate residual lesions, detect tumor recurrence earlier, diagnose and treat tumors, select personalized treatments, and monitor EC recurrence related to drug resistance (14).

The uterine cavity is connected to the vagina, and early EC often presents with abnormal vaginal bleeding as the initial symptom. Therefore, vaginal lavage fluid can accurately reflect the genetic changes in tumor tissue. Based on this, we retrospectively analyzed 11 FIGO stage IA EC patients. The vaginal lavage fluid of 11 patients was divided into vaginal lavage fluid supernatant and vaginal lavage fluid precipitate, which were used to extract circulating tumor DNA (Lavage fluid ctDNA) and sediment DNA (Lavage fluid sDNA). All specimens were subjected to molecular analysis using target NGS of 437 cancer-related genes. Fully evaluate the consistency of genomic distribution between tumor tissue and other different sample types, and determine whether vaginal lavage fluid testing may be a convenient method for early diagnosis of EC.

## 2 Materials and methods

### 2.1 Sample collection

11 patients with endometrial cancer were all patients who underwent surgery in the Department of Gynecology, Department 2, the Second Affiliated Hospital of Dalian Medical University between January 2023 and December 2023. All patients were aged 18–75 years, with no prior radiotherapy or chemotherapy before surgery and no history of other malignant tumors. Vaginal lavage fluid was obtained before curettage surgery, and subsequent pathological confirmation via curettage confirmed endometrial cancer in all cases. Preoperative imaging studies staged all patients as FIGO Stage IA. Following confirmation of FIGO Stage IA endometrial cancer, all patients underwent laparoscopic total hysterectomy with bilateral salpingo-oophorectomy and sentinel lymph node mapping and biopsy. This study was approved by the Ethics Committee of Second Affiliated Hospital of Dalian Medical University, and all patients provided informed consent forms. All samples were tested at Nanjing Shihe Medical Laboratory.

We collected 20ml of vaginal lavage fluid for each patient before surgery. The patient took the bladder lithotomy site and inserted a speculum into the vagina. 15ml of 0.9% sodium chloride injection was poured into the vagina for flushing. After flushing, the sterile syringe was used to extract the vaginal lavage fluid and placed in a 15ml sterile centrifuge tube.

Collect 8–10 milliliters of peripheral blood for each patient, and centrifuge plasma and white blood cells at 1800 rpm for 10 minutes as soon as possible after collection. The separated plasma is used for extracting ctDNA, while white blood cells are used as negative controls. And all 11 patients obtained formalin fixed tumor tissue specimens after surgery, all of which were confirmed qualified by experienced pathology experts.

To prepare ctDNA from vaginal lavage, we first removed cells from the vaginal lavage by low-speed centrifugation, followed by high-speed centrifugation to remove any debris. The resultant supernatant was then subjected to ctDNA extraction using the Qiagen QIAamp Circulating Nucleic Acid Kit (Qiagen, Shanghai, China).

### 2.2 Library preparation, sequencing and data analysis

Sequencing libraries were prepared using the KAPA Hyper Prep kit (KAPA Biosystems, MA, USA) according to the manufacturer’s suggestions for different sample types. In brief, 6.08–200 ng (median: 70.5 ng) of cfDNA or 1 μg of fragmented genomic DNA underwent end-repairing, A-tailing, and ligation with indexed adapters sequentially, followed by size selection using Agencourt AMPure XP beads (Beckman Coulter, FL, USA). Hybridization-based target enrichment was carried out with the GeneseeqOneTM pan-cancer gene panel (437 cancer-relevant genes), and xGen Lockdown Hybridization and Wash Reagents Kit (Integrated DNA Technologies). Captured libraries by Dynabeads M-270 (Life Technologies, MA, USA) were amplified in KAPA HiFi HotStart ReadyMix (KAPA Biosystems, MA, USA) and quantified by qPCR using the KAPA Library Quantification kit (KAPA Biosystems, MA, USA) for sequencing.

The libraries were paired-end sequenced on Illumina HiSeq4000 NGS platforms (Illumina, CA, USA) according to the manufacturer’s instructions. The mean coverage depth was > 100× for the whole blood control samples. For cfDNA samples, the original targeted sequencing depth was > 3000 ×. Trimmomatic was used for FASTQ file quality control (below 15 or N bases were removed). Reads were then mapped to the reference Human Genome (hg19) using Burrows-Wheeler.

Aligner (BWA-mem, v0.7.12) (https://github.com/lh3/bwa/tree/master/bwakit). Local realignment around the indels and base quality score recalibration was applied with the Genome. Analysis Toolkit (GATK 3.4.0) (https://software.broadinstitute.org/gatk/), which was also applied to detect germline mutations.VarScan2 was employed for somatic mutation detection. Somatic variant calls with at least 0.2% mutant allele frequency (MAF) and with at least 3 supporting-reads from both directions were retained. Common SNPs were filtered out using dbSNP (v137) and the 1000 Genomes database, followed by annotation using ANNOVAR. Genomic fusions were identified by FACTERA with default parameters. Copy number variations (CNVs) were detected using ADTEx (http://adtex.sourceforge.net) with default parameters. Somatic CNVs were identified using paired normal/tumor samples for each exon with the cut-off of 0.65 for copy number loss and 1.50 for copy number gain.

### 2.3 Statistical analysis

Quantitative data is expressed as median (range) or absolute (percentage). Use Wilcoxon non parametric testing to compare variables. Two-tailed P-value of<0.05 is considered statistically significant. All statistical analyses were conducted using R 4.3.2.

## 3 Results

### 3.1 Clinical and pathological data of endometrial cancer

A total of 11 patients with endometrial cancer in FIGO stage IA were included in this study. The median age was 52 years old, and the median BMI was 25.51kg/m2. Among them, 6 (6/11) patients had menopause, 10 (10/11) patients had abnormal vaginal bleeding, 8 (8/11) patients had moderate low differentiation, 3 (3/11) patients had high differentiation, of which 1 (1/11) patients had Lymphavascular tumor tumors. All patients did not have permanent depression, of which 4 (4/11) patients had hypertension, and 3 (3/11) patients had diabetes (**Table 1**).

**Table 1:** Clinical and pathological data of endometrial cancer.

Characteristics	N(%)/Value
Age, median(range), years	52(29-68)
Abnormal vaginal bleeding	
Yes	10(91%)
No	1(9%)
Differentiation	
Middle-Low	8(73%)
High	3(27%)
Lymphovascular tumor embolus	
Yes	1(9%)
No	10(91%)
Perineural invasion	
Yes	0(0)
No	11(100%)
Menopause	
Yes	6(55%)
No	5(45%)
High blood pressure	
Yes	4(36%)
No	7(64%)
Diabetes	
Yes	3(27%)
No	8(73%)
BMI, median(range), kg/m^2^	25.51(23.44-29.76)
CA125, median(range), U/ml	17.14(9.54-43)
CEA, median(range), ng/ml	0.84(0-2.25)
CA199, median(range), U/ml	21.58(0-84.05)
HE4, median(range), pmol/L	53.03(42.96-111.3)

BMI, body mass index; CA125, carbohydrate antigen 125; CEA, carcinoembryonic antigen; CA19-9, carbohydrate antigen 199; HE4, whey acidicprotein, wap.

### 3.2 Comparison of mutation abundance/MAF

**Figure 1:**
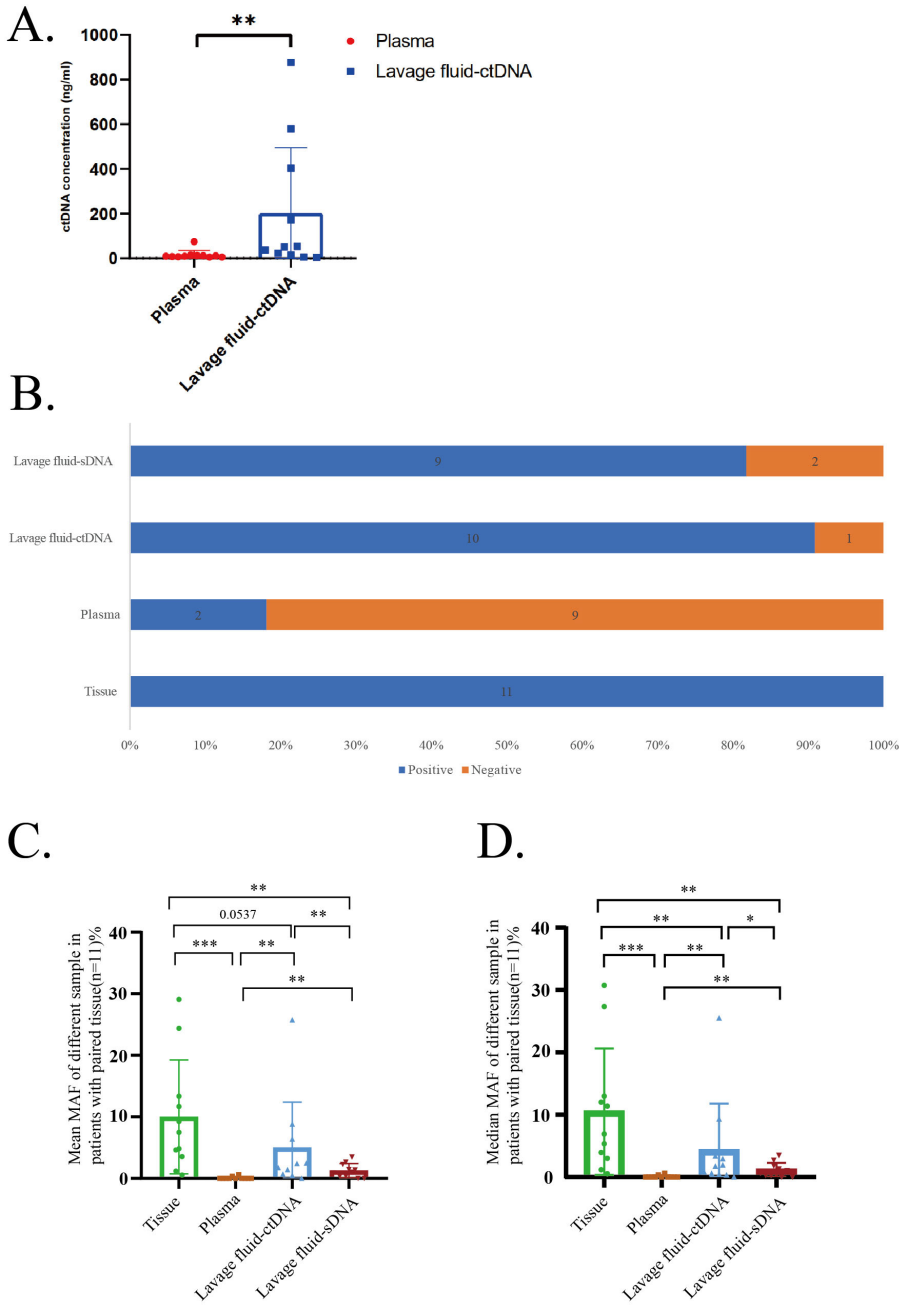
Comparison of mutation abundance/MAF. **(A)** ctDNA in different samples. **(B)** Positive detection rate in different samples. **(C)** Mean MAF of different sample in patients with paired tissue. **(D)** Median MAF of different sample in patients with paired tissue.

In 11 cases with matched tumor and blood tissues, the extracted ctDNA concentration was significantly higher in vaginal lavage fluid than in plasma (**Figure 1A**). We detected mutations in all tissue samples, with a positive detection rate of 100% (**Figure 1B**). The positive detection rate in vaginal lavage fluid precipitation was 81.8% (9/11), the supernatant of vaginal lavage fluid was 90.9% (10/11), and the positive detection rate in plasma was only 18.2% (2/11) (**Figure 1B**). The mean MAF of different sample in patients with paid trouble is significantly higher in tumor tissue than in plasma, vaginal lavage supernatant, and vaginal lavage fluid precipitate, and the mean MAF of different sample in patients with paid trouble is significantly higher in vaginal lavage fluid supernatant than in vaginal lavage fluid precipitate. The mean MAF of different sample in patients with paired tissues is also significantly higher in vaginal lavage fluid precipitate than in plasma. The same conclusion was reached in the median MAF of different samples in patients with paired issues (**Figures 1C, D**).

### 3.3 Comparison of genomic change types and consistency

#### 3.3.1 Genomic change detection for each type

**Figure 2:**
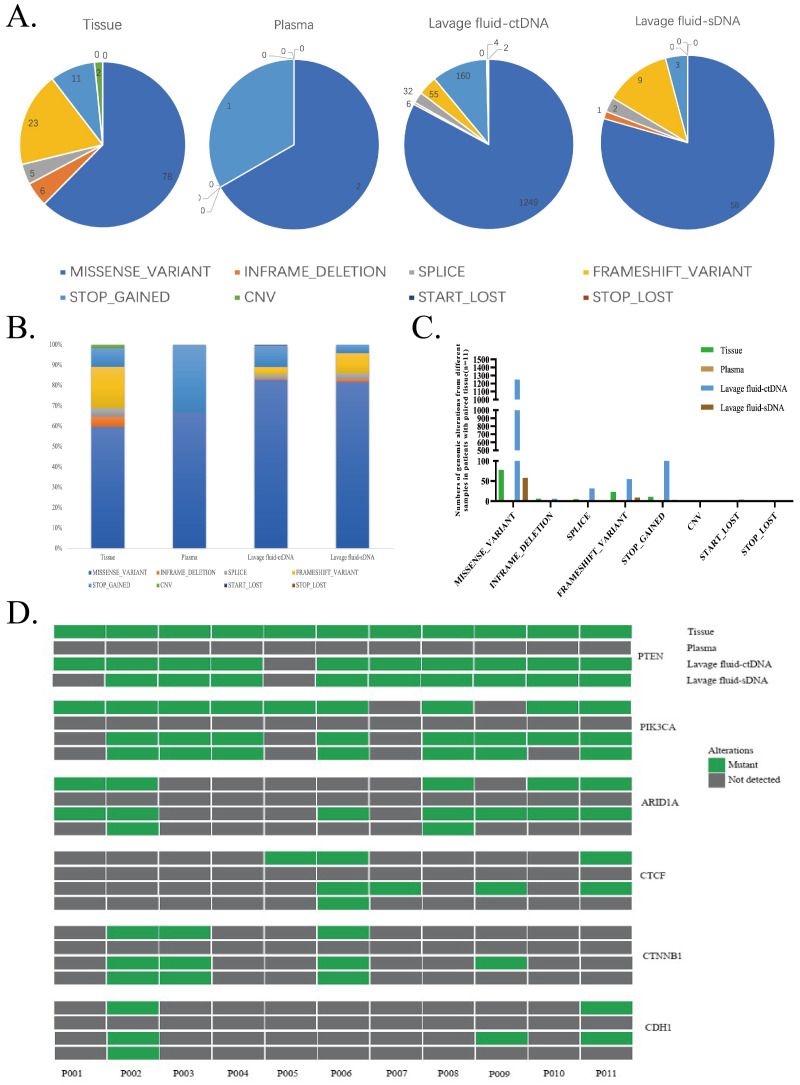
**(A)** Comparison of genomic change types and consistency. Genomic change detection for each type. **(B–D)** Comparison of genomic change consistency.

Among the 11 collected tumor tissue samples, changes were detected in 125 genomes of 95 genes, including Missense_Variant (78), Inframe_Deletion (6), Splice (5), Frameshift_Variant (23), Stop_Gained (11), and CNV (2). Among the 11 collected vaginal lavage supernatants, a total of 1508 genomic changes were detected in 420 genes, including Missense_Variant (1249), Inframe-Deletion (6), Splice (32), Frameshift_Variant (55), Stopp_Gained (160), Start_Lost (4), and Stop_Lost (2). In the precipitation of vaginal lavage fluid, changes were detected in 73 genomes of 55 genes, including Missense_Variant (58), Inframe_Deletion (1), Splice (2), Frameshift_Variant (9), and Stopp_Gained (3). However, in plasma tissue samples, we only detected changes in three genomes of three genes, including Missense_Variant (2) and Stop_Gained (1) (**Figure 2A**).

#### 3.3.2 Comparison of genomic change consistency

The main types of genomic changes in tumor tissue, vaginal lavage fluid supernatant, and vaginal lavage fluid precipitate are Misense variant. The types of genomic changes in the supernatant and sediment of vaginal lavage fluid are relatively consistent with those in tumor tissue samples, but significantly different from those in plasma. The number of Misense variants in the supernatant and sediment of vaginal lavage fluid is significantly higher than that in plasma (**Figures 2B, C**).

Among the six most frequently mutated genes in this study, the consistency between vaginal lavage supernatant and tumor tissue genomic changes was 81.8% (27/33), and the consistency between vaginal lavage precipitation and tumor tissue genomic changes was 66.7% (22/33) (**Figure 2D**).

### 3.4 The application of vaginal lavage fluid in the diagnosis of endometrial cancer

**Figure 3:**
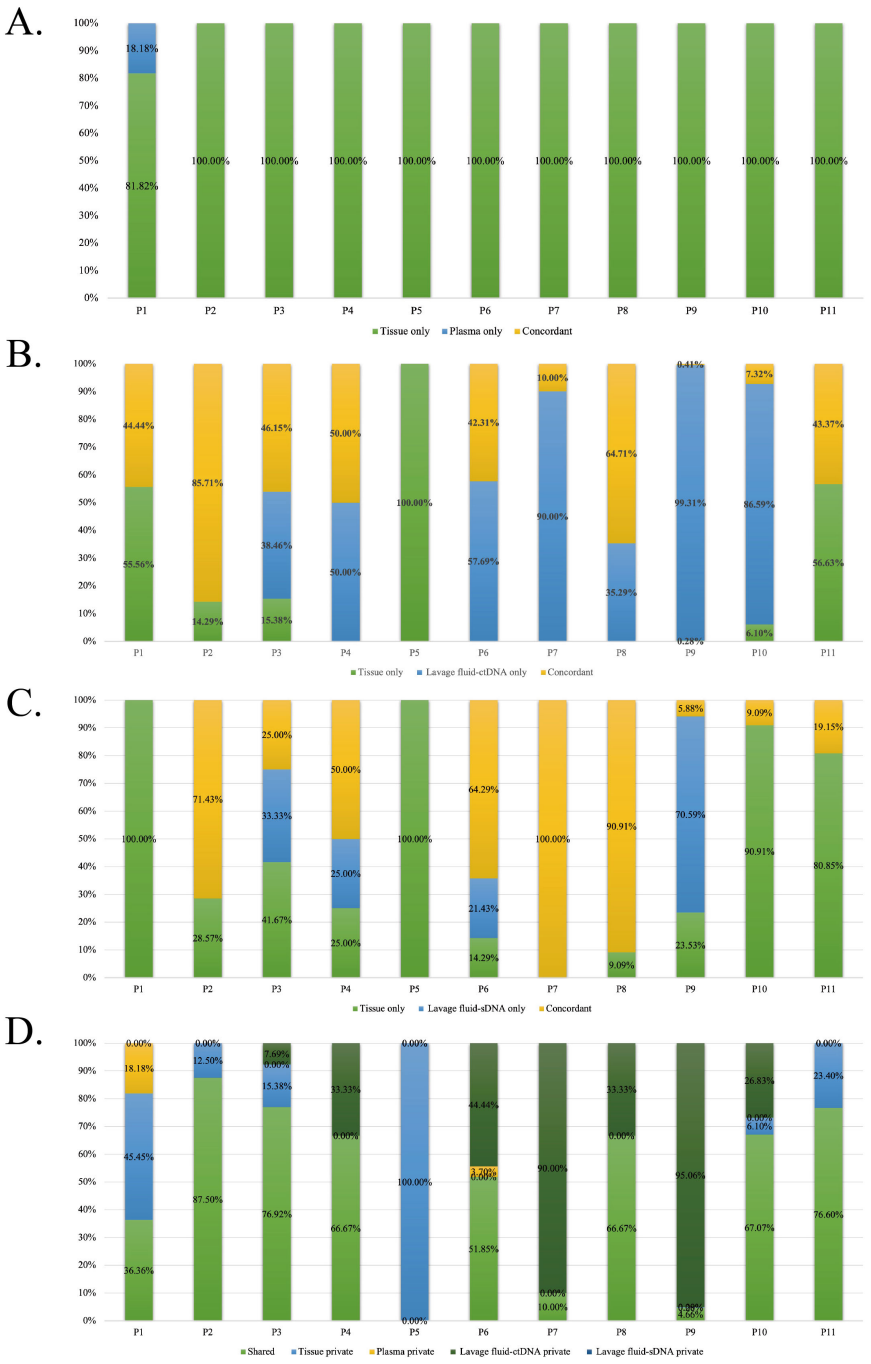
The consistency of genomic changes among the supernatant or precipitate of vaginal lavage fluid, tumor tissue and plasma. **(A)** The consistency of genomic changes among tumor tissue and plasma. **(B)** The consistency of genomic changes among tumor tissue and the supernatant of vaginal lavage fluid. **(C)** The consistency of genomic changes among tumor tissue and the precipitate of vaginal lavage fluid. **(D)** The consistency of genomic changes among the supernatant or precipitate of vaginal lavage fluid, tumor tissue and plasma.

According to the results of 11 EC patients who provided tumor tissue samples, the genomic changes detected between the supernatant or precipitate of vaginal lavage fluid and tumor tissue were significantly consistent with those detected between tumor tissue and plasma (**Figures 3A–C**). The consistency of genomic changes between the supernatant/precipitate of vaginal lavage fluid and tumor tissue was significantly higher than that between tissue and plasma (**Figures 3A–C**).

**Figure 4:**
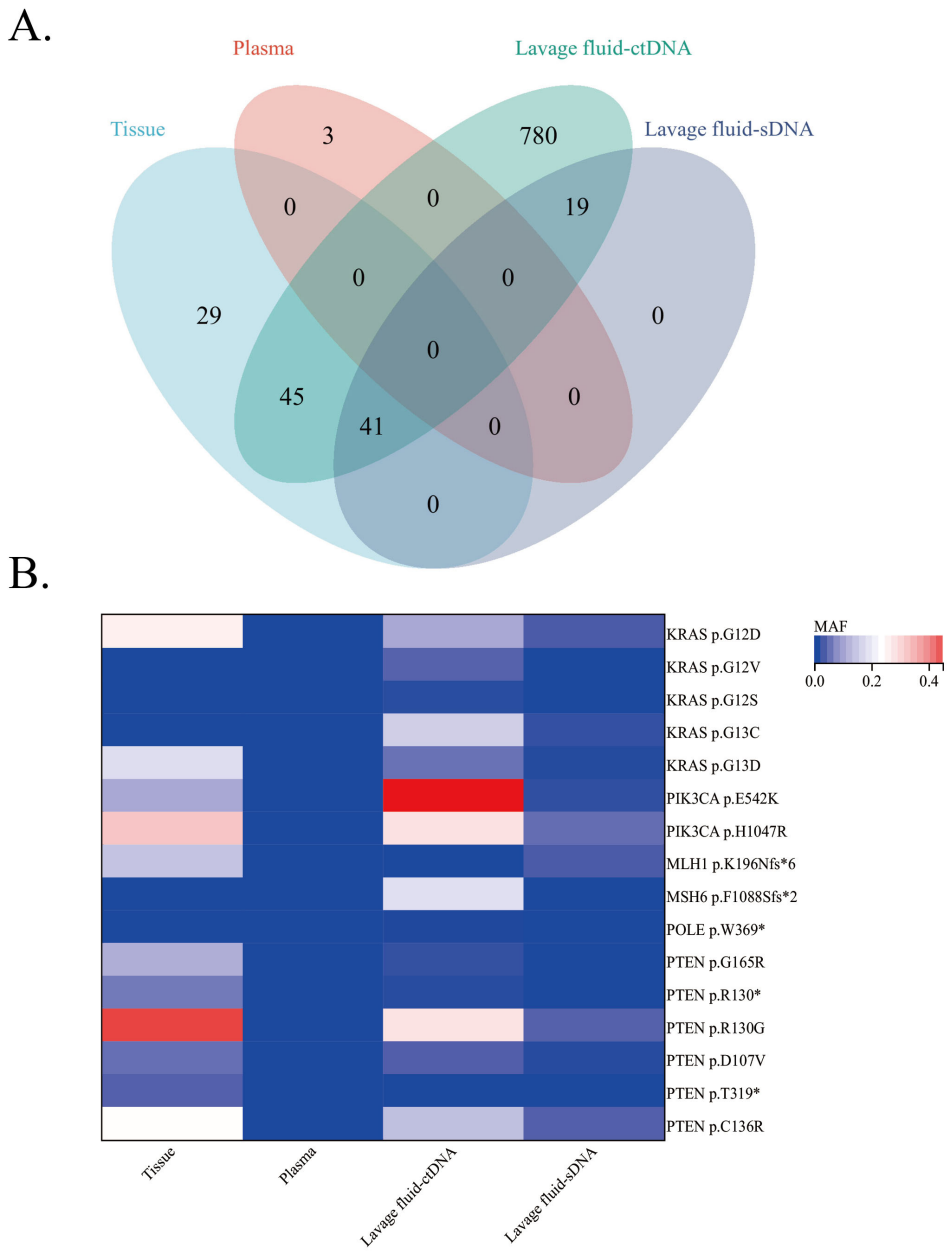
The application of vaginal lavage fluid in the diagnosis of endometrial cancer. **(A)** Quantity and consistency of genomic alterations detected in different samples. **(B)** Analysis of potential target genes for targeted therapy of endometrial cancer.

In addition, compared to mutations that cannot be detected in the plasma, additional genomic changes that were not found in tissues can be detected in the supernatant and sediment of vaginal lavage fluid in more patients (**Figures 3A–D**). The consistency of genomic changes between the supernatant and tumor tissue was 74.8% (86/115). There were no genomes present in tumor tissue, but an additional 799 genomic changes were found in the supernatant of vaginal lavage fluid (**Figure 4A**). The consistency between vaginal lavage fluid precipitation and tumor tissue was 35.7% (41/115). An additional 19 genomic changes were detected in the precipitate of vaginal lavage fluid, but not in tumor tissue, and these 19 genes can be detected in the supernatant of vaginal lavage fluid (**Figure 4A**).

Regarding potential targets for targeted treatment of endometrial cancer (KRAS, PIK3CA, MLH1, MSH6, POLE, PTEN), relevant genomic changes were also detected in the supernatant and sediment of vaginal lavage fluid (**Figure 4B**), and these genomic changes were more pronounced in the supernatant of vaginal lavage fluid.

## 4 Discussion

Early stage endometrial cancer usually has a good prognosis, while the 5-year survival rate for stage III endometrial cancer is only 50-60%, and the 5-year survival rate for stage IV endometrial cancer is only 15-17% (15) Due to the good prognosis of early endometrial cancer, early diagnosis of endometrial cancer is very important. Previous studies have shown that gene sequencing in biological fluids can detect tumor causing DNA, which may lead to early detection of tumors and achieve early diagnosis of EC (16).

The majority of patients with endometrial cancer experience abnormal vaginal bleeding as the initial symptom, with a small portion detected through ultrasound examination during physical examination. The main screening methods for suspected endometrial cancer are curettage and pathological examination. Curettage is an invasive procedure with significant pain and high screening costs. The uterine cavity is connected to the vagina, so we believe that endometrial cancer cells can detach from the vagina. Collecting vaginal lavage fluid for liquid biopsy can be a non-invasive and convenient method for early screening of endometrial cancer.

Therefore, we collected vaginal lavage fluid from patients suspected of having endometrial cancer prior to curettage, and subsequent pathological confirmation via curettage confirmed endometrial cancer in all cases. Preoperative imaging studies staged all patients as FIGO Stage IA. Following confirmation of FIGO Stage IA endometrial cancer, all patients underwent laparoscopic total hysterectomy with bilateral salpingo-oophorectomy and sentinel lymph node mapping and biopsy. Postoperative pathological diagnosis uniformly confirmed endometrioid adenocarcinoma, FIGO Stage IA. Finally, a total of 11 patients with FIGO Stage IA endometrial cancer were included in this study, with a median age of 52 years and a median BMI of 25.51kg/m2. Among them, 6 patients had menopause, 10 patients had abnormal vaginal bleeding, and only 1 patient was found during physical examination. Among them, 4 patients had hypertension and 3 patients had diabetes (**Table 1**). This is consistent with the first symptoms of endometrial cancer patients, and hypertension and diabetes are both high risk factors for endometrial cancer (17).

We performed molecular analysis on the supernatant of vaginal lavage fluid, vaginal lavage fluid precipitation, plasma, and tissues of all 11 patients using target NGS of 437 cancer-related genes. The study found that in 11 cases with matched tumor and blood tissues, the extracted ctDNA concentration was significantly higher in vaginal lavage fluid than in plasma (**Figure 1A**). And the positive detection rate in vaginal lavage fluid sediment and vaginal lavage fluid supernatant was significantly higher than that in plasma (**Figure 1B**). The mean and media MAF of different samples in patients with paid issues were significantly higher in the supernatant and precipitate of vaginal lavage fluid than in plasma (**Figures 1C, D**). This may be because the ctDNA in the plasma of stage IA endometrial cancer, as an early stage endometrial cancer, is very low. This also explains why the tumor markers in the plasma of early endometrial cancer patients did not show a significant increase. In the 11 patients studied, the median of CA125 was 17.14U/ml, CEA was 0.84ng/ml, CA199 was 21.58U/ml, and HE4 was 53.03pmol/L, all of which did not show a significant increase (**Table 1**).

Our further analysis found that the main type of genomic change in tumor tissue, vaginal lavage fluid supernatant, and vaginal lavage fluid precipitate is Misense variant. The types of genomic changes in the supernatant and sediment of vaginal lavage fluid were relatively consistent with those in tumor tissue samples, and the number of Misense variants in the supernatant and sediment of vaginal lavage fluid was significantly higher than that in plasma (**Figures 2B, C**). In addition, among the six most frequently mutated genes in this study, the consistency between genomic changes in vaginal lavage fluid supernatant and tumor tissue was 81.8%, and the consistency between genomic changes in vaginal lavage fluid sediment and tumor tissue was 66.7% (**Figure 2D**). This indicates that vaginal lavage fluid has a significant advantage over plasma in the early diagnosis of EC.

According to the results of 11 EC patients who provided tumor tissue samples, the genomic changes detected between the supernatant or precipitate of vaginal lavage fluid and tumor tissue were significantly consistent with those detected between tumor tissue and plasma (**Figures 3A–C**). The consistency of genomic changes between the supernatant/precipitate of vaginal lavage fluid and tumor tissue was significantly higher than that between tissue and plasma (**Figures 3A–C**). In addition, the consistency of genomic changes between the supernatant of vaginal lavage fluid and tumor tissue was 74.8%, and an additional 799 genomic changes were detected in the supernatant of vaginal lavage fluid. The consistency between vaginal lavage fluid precipitation and tumor tissue was 35.7%. An additional 19 genomic changes were detected in the precipitate of vaginal lavage fluid (**Figure 4A**).

In order to further clarify the indicative effect of vaginal lavage fluid on potential targets for endometrial cancer, we selected 6 potential targets for endometrial cancer treatment. They are KRAS, PIK3CA, MLH1, MSH6, POLE, PTEN, respectively. KRAS mutations can cause resistance to EGFR inhibitors, therefore, the KRAS mutation status is directly related to chemotherapy resistance. The KRAS mutation in early type I EC patients may provide important information for prognostic stratification and further provide personalized treatment options (18) The PI3K/AKT pathway is activated in various cancers and is associated with chemotherapy resistance, and approximately 25-53% of cases in EC have PIK3CA mutations (19, 20) Compared with mismatch repair deficiency (MMR-D) EC, MLH1 promoter hypermetabolism EC may constitute a unique clinical pathological entity with therapeutic significance (21) Low expression of MSH6 can serve as a potential biomarker for predicting better prognosis, active immune status, higher levels of immune checkpoint expression, and better response to immune checkpoint inhibitors in EC. The pathogenic cellular mutations of the POLE gene usually occur early and are related to the occurrence of endometrial cancer (22) The ubiquitination and degradation of PTEN protein in endometrial cancer have long been proven (23) PTEN is a tumor suppressor gene involved in the PI3K-PTEN-AKT mTOR pathway, and its somatic mutations occur in 69% -80% of EECs (24) Moreover, PTEN mutations occur in the early stages of type I EC tumor progression, and the absence of PTEN in endometrial hyperplasia is significantly associated with an increased risk of EC (23, 25, 26) As these six gene mutations are all important in early endometrial cancer, we analyzed these six gene mutations in vaginal lavage fluid and detected related genomic changes in the supernatant and sediment of vaginal lavage fluid (**Figure 4B**). This genomic change is more pronounced in the supernatant of vaginal lavage fluid. So, compared to plasma, vaginal lavage fluid is more suitable as an indicator of potential targets, but it still needs to be based on pathology. If the patient cannot tolerate curettage and curettage pathology, vaginal lavage fluid can be considered as a basis for targeted treatment.

However, there are still certain limitations to the research. Currently, the sample size of the study is slightly small. Therefore, we will verify our conclusions through a larger sample size.

## Data Availability

The original contributions presented in the study are included in the article/supplementary material. Further inquiries can be directed to the corresponding author.
